# Early Placement of Patient‐Specific One‐Piece Drill‐Free Fully Digital Designed Dental Implants: A Feasibility Study

**DOI:** 10.1111/jcpe.14204

**Published:** 2025-07-20

**Authors:** Giuseppe Alexandre Romito, Mohamed Ahmed Hassan, Marina Nogueira de Castro Galvão Corrente, Julia Dahdal Aoun, Vitor Marques Sapata, Roger Nishyama, Cristina Cunha Villar

**Affiliations:** ^1^ Discipline of Periodontics, School of Dentistry University of São Paulo São Paulo Brazil; ^2^ Discipline of Prosthodontics, School of Dentistry University of São Paulo São Paulo Brazil

**Keywords:** computer‐aided design, computer‐assisted manufacturing, cone‐beam computed tomography, dental implants, tooth root

## Abstract

**Aim:**

CBCT and CAD/CAM technologies have allowed the development of patient‐specific implants requiring no drilling. This prospective study evaluated the safety and effectiveness of a novel, drill‐free, one‐piece root‐analogue implant designed using a digital workflow and manufactured additively.

**Materials and Methods:**

Patients with non‐restorable maxillary anterior or premolar teeth requiring extraction and implant placement were included. Implant design was based on pre‐extraction cone‐beam computed tomography (CBCT) and optical impressions of the target tooth, adjacent teeth and opposing dentition, and post‐extraction scanning of the extracted tooth. Within 14 days post extraction, the implant was designed, manufactured and placed into the alveolar socket, and a temporary crown was installed out of occlusion. The final crown was installed after 3 months. Clinical parameters, including plaque index, gingival index, bleeding on probing, suppuration, peri‐implant mucosal margin position, probing depth, probing depth relative to the implant platform and keratinised tissue, were collected at 3, 6, 9, 12 and 24 months post loading. Radiographic measurements as well as patient‐ and clinician‐reported outcomes were also assessed.

**Results:**

The study cohort comprised 12 patients. The cumulative implant survival and success rates reached 100% and 90%, respectively. Clinical and radiographic parameters consistently indicated healthy peri‐implant tissues. Patient‐reported outcomes demonstrated high satisfaction and minimal discomfort. Visual analogue scale (VAS) scores for overall satisfaction remained high, with a median of 10 at final restoration delivery (95% CI: 10.00–10.00) and at the 24‐month follow‐up (95% CI: 9.70–10.01).

**Conclusion:**

These patient‐specific, root‐analogue implants demonstrated both safety and effectiveness, along with high patient satisfaction rates up to 24 months post loading.

## 1 Introduction

Implant success depends on multiple factors, including clinician expertise, bone volume, implant design, surgical technique and insertion torque (Roccuzzo et al. 2022; Zoghbi et al. 2011; Kochar et al. 2022; Moraschini et al. 2015; Chatzopoulos and Wolff 2023). Conventional drilling may cause intraoperative discomfort and prolong recovery (Kahn et al. 2021; Maglione et al. 2019; Jain et al. 2024). Despite surgical advances, implant macro‐design has seen little change.

Digital tools such as cone‐beam computed tomography (CBCT), computer‐aided design/computer‐aided manufacturing (CAD/CAM) and additive manufacturing have enhanced implant precision and enabled the development of drill‐free, patient‐specific implants (Putra et al. 2022; Ochandiano et al. 2022; Bidra et al. 2013). These CAD/CAM‐designed root‐analogue implants fit the extraction socket precisely (Mangano et al. 2014; Moin et al. 2018; Evans et al. 2018; Westover 2019), improving 3D positioning and minimising gaps, potentially reducing the need for bone regeneration (Dantas et al. 2021; Regish et al. 2013). By avoiding drilling, they preserve the bone, enhance primary stability (Regish et al. 2013; Chen et al. 2014) and simplify prosthetic workflows (Liu et al. 2022).

However, the safety and performance data remain limited (Mangano et al. 2014; Moin et al. 2018; Liu et al. 2022), and failures have been linked to inaccuracies from CBCT‐only planning (Moin et al. 2018). Thus, this study aimed to evaluate the safety and clinical effectiveness of a novel drill‐free, one‐piece CAD/CAM‐designed root‐analogue implant, engineered using combined CBCT and STL data.

## 2 Materials and Methods

This single‐centre, prospective, non‐randomised study was conducted at the University of São Paulo, Brazil, from February 2022 to November 2024. The primary aim was to evaluate 12‐month post‐loading implant survival. The study followed the 2008 Helsinki Declaration, was registered (RBR‐848ygb3) and approved by the institutional ethics committee (no. 5.509.540). A conservative sample of 12 patients was enrolled. All participants provided informed consent (flowchart in Figure S1).

Eligible patients required extraction of a single‐rooted tooth with immediate implant placement. Inclusion criteria were age 22–75 years, unrestorable tooth, adequate bone and soft tissue at the site (Socket Type 1A on CBCT) and no apical or peri‐radicular pathology. Exclusion criteria included surgical/systemic contraindications, untreated periodontal diseases, poor hygiene, parafunction, certain medical conditions (e.g., diabetes, cancer, HIV, metabolic bone disease), recent immunosuppressant use, bisphosphonates, radiation, pregnancy, smoking or concurrent dental/clinical trials.

### 2.1 Additional Exclusion Criteria at Tooth Extraction

- Buccal plate < 1 mm or fractured.
- Root fracture or missing socket walls.
- Socket infection or communication with vital structures.
- Incompatible root/alveolus shape or ostectomy.

### 2.2 At Implant Placement

- Local complications (infection, trauma, etc.).
- Medical/dental history changes.
- Implant not placed within 14 days of extraction.

### 2.3 Implant Design and Manufacturing

#### 2.3.1 Imaging Acquisition

CBCT images were acquired (Carestream CS 9300 system, Carestream, Rochester, USA) before tooth extraction (isotropic voxel size = 160 μm, 8 × 8 cm^2^ field of view, tube voltage of 74 kV, tube current of 12 mA, 14‐bit resolution, 360° rotation completed in 16 s). The acquired data were stored in DICOM format and analysed using the Mimics software (v. 24, Materialise, Leuven, Belgium).

Optical impressions of the subject tooth as well as the adjacent and opposing dentition were taken using a scanner (TRIOS 3, 3Shape, København, Copenhagen) prior to tooth extraction. On the day of the extraction, the extracted tooth was also scanned.

#### 2.3.2 Image Treatment and Implant Manufacturing

CBCT imaging slices were used to construct a 3D model. The subject tooth was segmented using the software (Mimics). The resultant model along with optical scan models and tooth scan were merged (Mimics) to design the implant.

The implant surface consisted of an uncoated titanium alloy with a surface roughness of 100 μm. The lower portion of the implant featured a lattice macro‐surface with an average nominal depth ranging from 300 to 600 μm and a porosity of 50%–70%. The coronal portion of the implant root incorporated six circumferential micro‐grooves, each 0.1 mm deep. The implant was designed to fit precisely within the mesial‐distal space of the extraction socket, with a reduced buccal‐lingual/palatal dimension (0.1–0.2 mm smaller than the original tooth root) to minimise excessive pressure on the buccal plate. The transgingival portion was engineered with a high‐polish finish, while the emergence profile and abutment were designed to optimise soft‐tissue support.

The implant was fabricated using a metal powder bed fusion process out of Grade 23 Titanium alloy (Ti 6Al4V ELI). Once printed, the implants underwent processes to achieve their final specifications for material strength, surface finish, cleanliness and sterility. Implants ranged from 9.32 to 17.00 mm in length, with an average of 13.86 ± 2.06 mm.

### 2.4 Surgical and Prosthetic Procedures

#### 2.4.1 Dental Extraction

**FIGURE 1:**
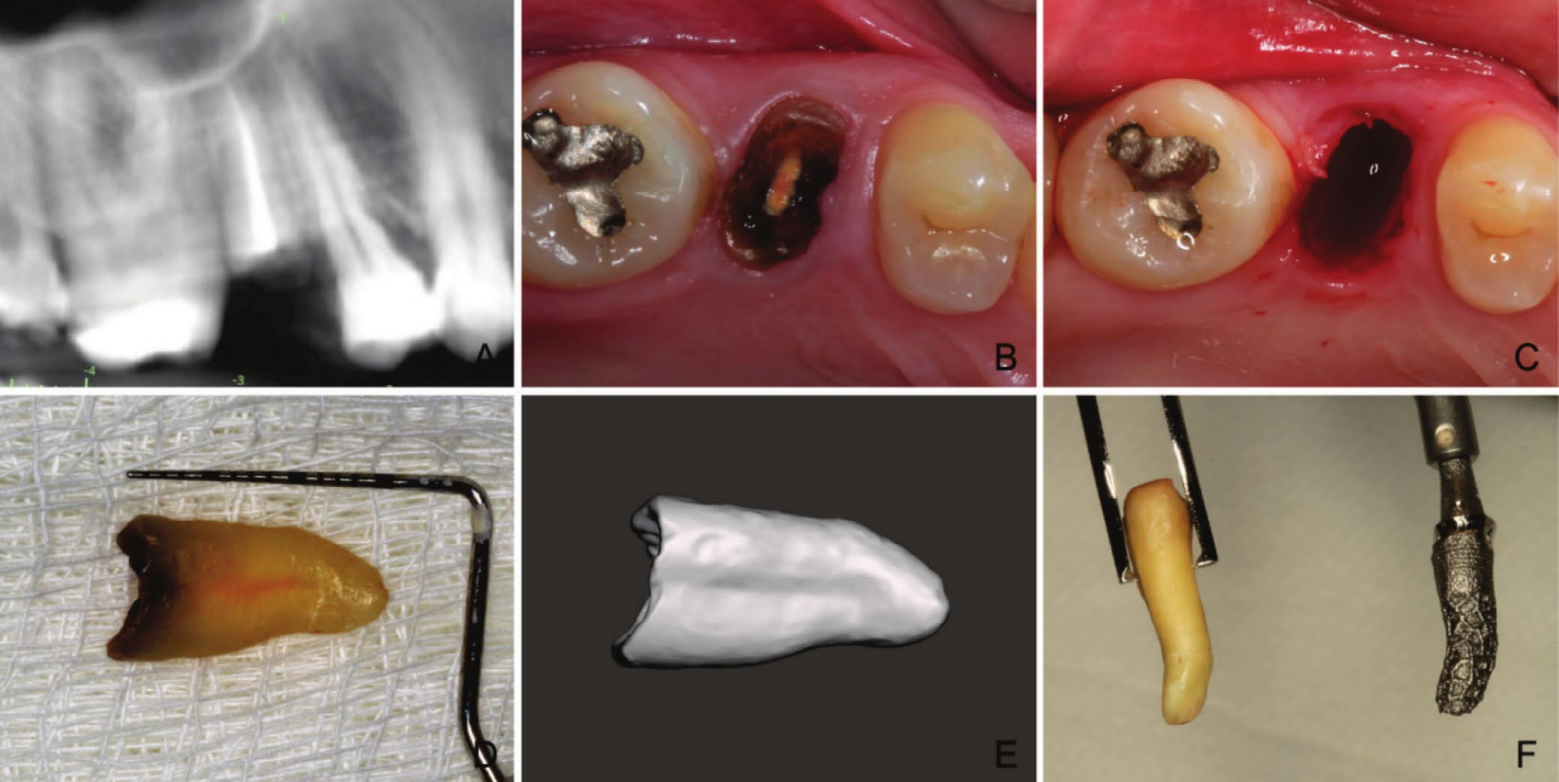
Extracted root and root‐analogue implant design. (A) Preoperative 3D tomography. (B) Residual root before extraction (C) Extraction site. (D) Extracted root. (E) Extracted root scan. (F) Extracted root and the corresponding root‐analogue implant.

Patients rinsed with 0.12% chlorhexidine before flapless tooth extraction under local anaesthesia (Figure 1). After debridement and socket inspection, a collagen sponge was placed and secured with 4.0 nylon ‘figure‐8’ or horizontal mattress sutures. A retainer was used for protection and aesthetics. Sutures were removed within 14 days.

### 2.5 Implant Placement and Immediate Provisionalisation

**FIGURE 2:**
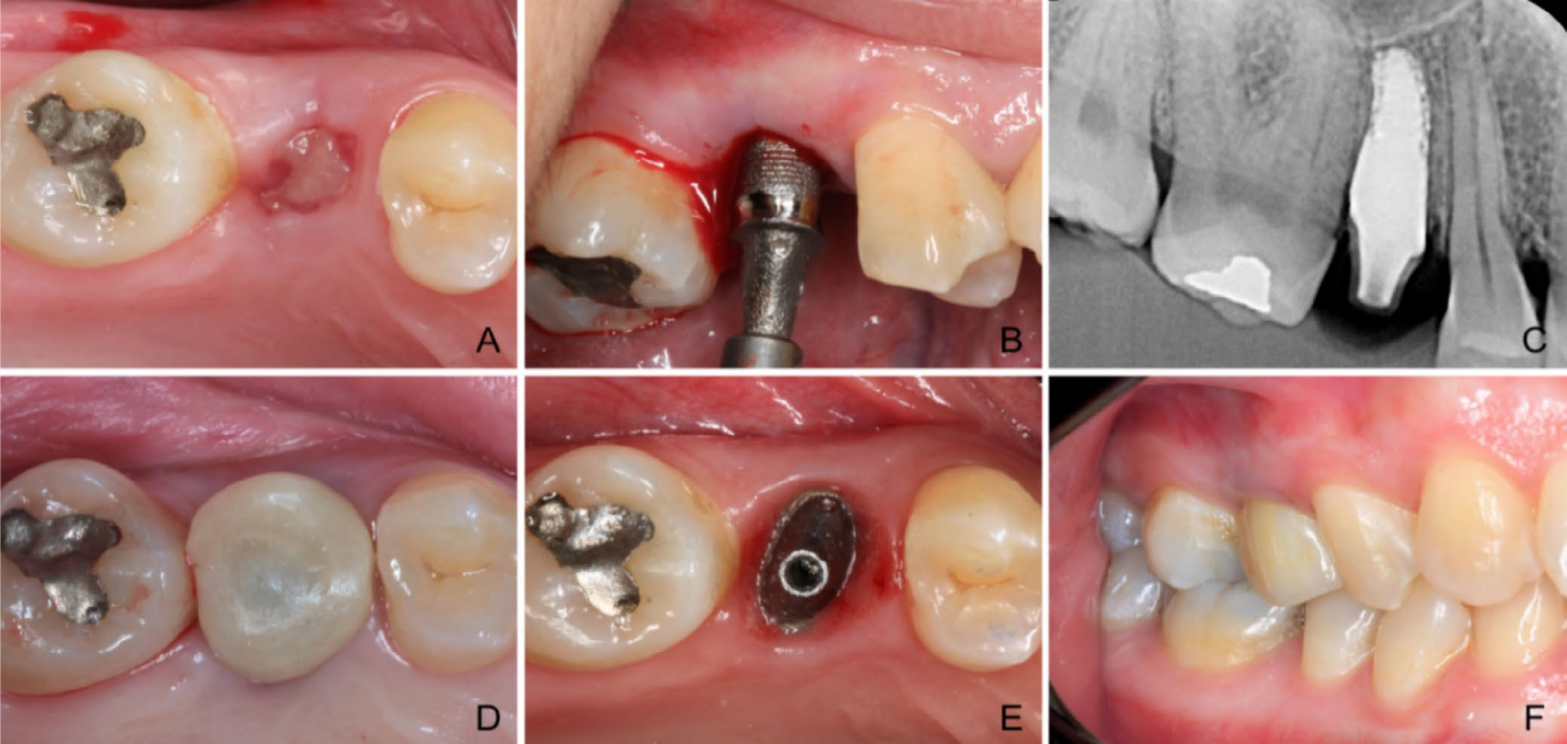
Root‐analogue implant‐based rehabilitation. (A) 2 weeks after extraction. (B) Implant placement (C) Periapical x‐ray immediately after implant placement (D) Temporary crown (E) Implant impression after 3 months (F) Definitive crown delivery.

All procedures were performed by a single experienced surgeon (G.A.R.). After local anaesthesia, the socket was exposed, debrided, irrigated and de‐epithelialised to prevent soft‐tissue ingrowth. Bleeding was induced to promote healing. Implants were manually positioned (using a driver attached to the implant), gently pressed into the socket and subsequently tapped into place using a surgical mallet. Clinically, it becomes evident that once the correct positioning is achieved, tapping produces an audible change from a higher pitched sound to a lower pitched one. Buccal orientation was verified via the implant's dimple mark. Primary stability was assessed tactilely, and radiographs confirmed proper platform alignment with the alveolar crest (Figure 2C). Immediate provisional crowns were placed out of occlusion. Postoperative pain was managed with dipyrone.

### 2.6 Final Restoration

Three months post implant placement, an intraoral scan was performed without crown seat preparation. The scan was matched to the manufacturer's 3D implant model, and final zirconia (3Y‐TZP) crowns were CAD/CAM‐designed, milled and cemented with resin cement. Radiographs confirmed accurate fit and no excess cement.

### 2.7 Follow‐Up Visits and Outcome Assessments

Follow‐up assessments were conducted at 3 days, 2 weeks and 3 months after implant placement, with subsequent visits at 3, 6, 9, 12, and 24 months post final restoration. In addition, the following parameters were recorded by a single trained and calibrated investigator (M.A.H.).

### 2.8 Safety Assessment

Safety evaluations were conducted throughout the study, monitoring all device‐ and procedure‐related adverse events.

### 2.9 Clinical Parameters

Clinical data were collected at six sites per implant (except KT, assessed mid‐buccally only). The valuated parameters included the following:

- O'Leary Plaque Index(O'Leary et al. 1972).
- Modified Gingival Index (mGI) (Mombelli et al. 1987).
- Bleeding on probing (BOP) and suppuration (dichotomous, within 30 s).
- Probing depth (PD) measured from the peri‐implant mucosal margin to the bottom of the sulcus/pocket.
- Mucosal margin position (MM) relative to implant shoulder (negative if subgingival).
- Probing depth relative to the implant platform (PD‐IP) measured from the bottom of the sulcus/pocket to the implant shoulder.
- KT width measured from the muco‐gingival junction to the peri‐implant mucosal margin.
- Mobility (MOB) assessed dichotomously through manual palpation using a blunt‐ended instrument.

### 2.10 Radiographic Measurements

Crestal bone changes were measured at 3, 6, 9, 12 and 24 months post restoration using digital periapical radiographs with customised bite blocks. ImageJ software (National Institutes of Health, USA) was used for the analysis. Measurements included total implant length and mesial/distal bone levels relative to the implant–abutment junction (IAJ) (Figure S2) (Yoo et al. 2006). Manufacturer‐reported implant lengths were used for calibration. And to account for magnification errors, a correction factor was applied using the formula

$$\begin{array}{c} \text{correction factor}=\text{actual implant length}/\\ \;\text{measured implant length} \end{array}$$

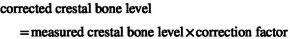




### 2.11 Implant Survival, Implant Success and Diagnosis

Implants were considered successful when they were free of persistent pain, infection, bone loss, mobility, radiolucency, neuropathy or paresthesia (Albrektsson et al. 1986). Survival was defined as the implant's physical presence in the oral cavity, regardless of function. Diagnoses followed the World Workshop definitions for peri‐implant health, mucositis and peri‐implantitis (Renvert et al. 2018).

### 2.12 Facial Peri‐Implant Marginal Mucosa Stability

Facial peri‐implant mucosa stability was assessed using sequential intraoral scans analysed via 3Shape's monitoring software. Baseline scans at crown delivery were compared with 12‐ and 24‐month follow‐ups. Models were aligned using a best fit algorithm, and vertical mucosal changes at the mid‐facial aspect were measured.

### 2.13 Patient‐ and Clinician‐Reported Outcomes

Patient‐reported outcomes (PROs) were assessed using a 10‐point scale across three domains: discomfort, aesthetics and satisfaction. Discomfort was evaluated before extraction, at 3 days, 2 and 4 weeks post implant placement, at final crown delivery and at 3, 6, 9, 12 and 24 months after crown placement. Aesthetic satisfaction and overall satisfaction were assessed at the pre‐extraction visit, final crown delivery and 3, 6, 9, 12, and 24 months post crown placement. Clinician satisfaction scores were recorded for the time required, ease of use and aesthetics.

### 2.14 Statistical Analysis

Continuous variables were summarised using descriptive statistics. Categorical variables were summarised by absolute and relative frequencies. The Shapiro–Wilk test was used to evaluate data distribution normality. Statistical analysis was conducted using repeated‐measures ANOVA with post hoc Tukey tests for parametric data and Friedman test with post hoc Durbin–Conover tests for non‐parametric data. Statistical significance was set at *p* < 0.05. All statistical analyses were performed using Jamovi software version 2.3.21.

## 3 Results

**FIGURE 3:**
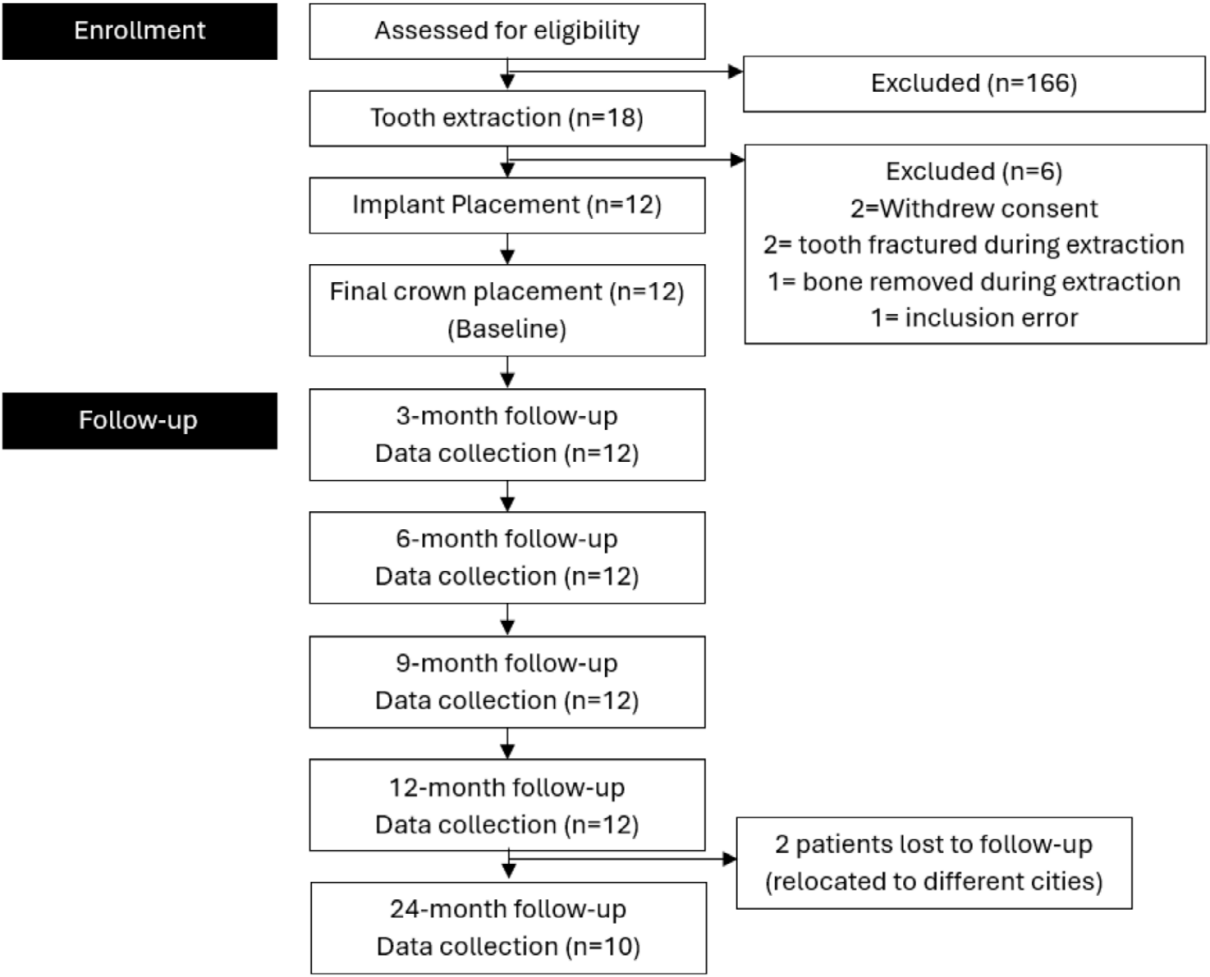
Flow diagram of patient recruitment.

A total of 184 patients were screened, and 18 patients underwent single‐rooted tooth extraction. Six patients were excluded because of exclusion criteria or consent withdrawal, leaving 12 patients (10 females, 2 males; average age 38.58 ± 9.26 years) for analysis (Figure 3). Details of the tooth extraction and implant sites are given in Table 1.

**TABLE 1:** Treated sites, implant survival and safety outcomes.

Patient	Region (tooth)	Indication for tooth extraction	Implant survival	Safety outcome
1	15	Caries	Yes	No AE
2	45	Caries	Yes	No AE
3	15	Caries	Yes	Suppuration
4	15	Caries	Yes	No AE
5	25	Caries	Yes	Suppuration
6	15	Caries	Yes	No AE
7	25	Caries	Yes	No AE
8	15	Caries	Yes	Suppuration
9	35	Caries	yes	No AE
10	21	Fracture	Yes	No AE
11	21	Failed endodontic treatment	Yes	No AE
12	35	Caries	Yes	Pre‐loading mobility

Abbreviation: AE, adverse event.

### 3.1 Safety Outcomes

None of the patients experienced pain, bleeding, swelling or infections postoperatively. Suppuration was observed in three patients at 3‐, 6‐ and 24‐month evaluations, which were resolved with mechanical scaling. One implant exhibited mobility prior to restoration, extending the time to final restoration to 6 months for osseointegration. No implant required repositioning or adjustment.

### 3.2 Clinical and Radiographic Outcomes

Clinical and radiographic parameters are summarised in Table 2.

**TABLE 2:** Clinical outcomes.

Clinical parameters		Final restoration (baseline) (*n* = 12)	3 months post loading (*n* = 12)	6 months post loading (*n* = 12)	9 months post loading (*n* = 12)	12 months post loading (*n* = 12)	24 months post loading (*n* = 10)
Success	%	100%	91.67%	83.33%	100%	100%	90%
PI	Mean (SD)	0.12 (0.25)	0.23 (0.30)	0.18 (0.31)	0.11 (0.16)	0.12 (0.14)	0.25 (0.28)
	Median (95% CI)	0.00^a^ (0.03–0.28)	0.17^a^ (0.04–0.43)	0.00^a^ (0.02–0.38)	0.00^a^ (0.01–0.21)	0.08^a^ (0.03–0.21)	0.33^a^ (0.10–0.57)
mGI	Mean (SD)	1.45 (0.43)^ab^	1.82 (0.39)^a^	1.84 (0.53)^a^	1.46 (0.48)^ab^	1.29 (0.32)^b^	1.60 (0.40)^ab^
	Median (95% CI)	1.50 (1.18–1.73)	1.75 (1.57–2.07)	1.75 (1.51–2.18)	1.42 (1.15–1.76)	1.33 (1.09–1.49)	1.75 (1.46–1.94)
BOP	Mean (SD)	0.47 (0.29)^ab^	0.59 (0.16)^a^	0.65 (0.32)^ab^	0.40 (0.25)^ab^	0.33 (0.21)^b^	0.41 (0.21)^ab^
	Median (95% CI)	0.50 (0.29–0.66)	0.67 (0.49–0.70)	0.67 (0.45–0.85)	0.42 (0.24–0.56)	0.33 (0.19–0.46)	0.50 (0.22–0.61)
SUP	Mean (SD)	0.00 (0.00)	0.04 (0.10)	0.06 (0.15)	0.00 (0.00)	0.00 (0.00)	0.05 (0.16)
	Median (95% CI)	0.00^a^ (0.00–0.00)	0.00^a^ (0.02–0.11)	0.00^a^ (0.04–0.15)	0.00^a^ (0.00–0.00)	0.00^a^ (0.00–0.00)	0.00^a^ (0.06–0.16)
PD (mm)	Mean (SD)	2.84 (0.67)^ab^	3.33 (0.58)^ab^	3.62 (0.95)^a^	3.19 (0.76)^ab^	2.86 (0.90)^b^	3.07 (0.84)^ab^
	Median (95% CI)	2.75 (2.42–3.26)	3.16 (2.81–3.57)	3.67 (3.01–4.23)	3.08 (2.71–3.68)	2.83 (2.29–3.43)	3.10 (2.48–3.66)
MM (mm)	Mean (SD)	−1.15 (1.14)	−1.58 (1.20)	−1.99 (1.32)	−1.91 (1.24)	−1.03 (1.80)	−1.20 (1.24)
	Median (95% CI)	−1.42^b^ (−1.87 to −0.43)	−1.75^ab^ (−2.34 to −0.82)	−2.17^a^ (−2.82 to −1.15)	−2.25^a^ (−2.70 to −1.13)	−0.83^ab^ (−2.17–0.11)	−1.67^b^ (−1.87 to −0.43)
Buccal MM (mm)	Mean (SD)	−0.67 (1.07)	−0.83 (1.11)	−1.08 (1.38)	−1.25 (1.36)	−0.67 (1.37)	−0.5 (1.58)
	Median (95% CI)	−1.0^a^ (−1.35–0.02)	−1.0^a^ (−1.54 to −0.13)	−1.0^a^ (−1.96 to −0.21)	−1.0^a^ (−2.11 to −0.39)	−0.5^a^ (−1.54–0.20)	−0.5^a^ (−1.63–063)
Buccal MM recession ≥ 1 mm (n)		2	1	2	1	3	3
PD‐IP (mm)	Mean (SD)	1.69 (1.14)	1.75 (0.97)	1.64 (1.03)	1.27 (1.02)	1.84 (1.43)	1.85 (0.92)
	Median (95% CI)	1.50^ab^ (0.97–2.41)	1.42^a^ (1.13–2.37)	1.33^ab^ (0.98–2.29)	0.92^b^ (0.63–1.92)	2.08^ab^ (0.94–2.75)	1.85^ab^ (0.83–2.80)
Buccal PD‐IP (mm)	Mean (SD)	1.25 (0.87)	1.50 (1.09)	1.17 (0.94)	0.92 (1.00)	1.42 (1.31)	1.40 (1.27)
	Median (95% CI)	1.00^a^ (0.70–1.80)	1.00^a^ (0.81–2.19)	1.00^a^ (0.57–1.76)	1.00^a^ (0.28–1.55)	1.50^a^ (0.58–2.25)	1.00^a^ (0.50–2.30)
Buccal KT (mm)	Mean (SD)	4.00 (2.37)	4.08 (2.39)	3.92 (2.15)	3.92 (2.19)	4.00 (2.09)	3.60 (2.17)
	Median (95% CI)	4.00^a^ (2.49–5.51)	4.50^a^ (2.56–5.60)	4.50^a^ (2.55–5.28)	4.00^a^ (2.52–5.31)	4.50^a^ (2.67–5.33)	4.00^a^ (2.05–5.15)
Mobility (n)	Implants with mobility	0	0	0	0	0	0
Mesial bone level (mm)	Mean (SD)	2.43 (1.42)	2.46 (1.44)	2.48 (1.52)	2.56 (1.60)	2.62 (1.80)	3.28 (1.44)
	Median (95% CI)	2.49^a^ (1.53–3.32)	2.52^a^ (1.55–3.38)	2.49^a^ (1.51–3.44)	2.48^a^ (1.55–3.58)	2.18^a^ (1.47–3.76)	2.97^a^ (2.25–4.30)
Distal bone level (mm)	Mean (SD)	2.38 (1.29)	2.47 (1.46)	2.63 (1.55)	2.88 (1.72)	2.96 (1.85)	3.25 (1.21)
	Median (95% CI)	2.81^a^ (1.56–3.20)	2.40^a^ (1.54–3.40)	2.55^a^ (1.64–3.61)	3.16^a^ (1.78–3.97)	3.29^a^ (1.78–4.14)$	3.17^a^ (2.39–4.12)
Digital MM changes	Mean (SD)	N/A	N/A	N/A	N/A	0.31 (0.32)	0.34 (0.37)
	Median (95% CI)	N/A	N/A	N/A	N/A	0.18^a^ (0.11–0.51)	0.2^a^ (0.07–0.60)

*Note*: Statistical differences are indicated by different letters and were determined using repeated measures ANOVA with a post hoc Tukey test for parametric data and Friedman with a post hoc Durbin–Conover test for nonparametric data.

Abbreviations: BOP, bleeding on probing; KT, keratinized tissue; mGI, modified gingival index; MM, position of the mucosal margin; N/A, not applicable; PD, probing depth; PD‐IP, probing depth relative to the implant platform; PI, plaque index.

#### 3.2.1 Survival and Success

All implants survived; 90% were successful at 24 months. No peri‐implantitis was observed. Mucositis occurred in two implants at 3 and 6 months and in one implant at 24 months. The remaining implants remained healthy.

#### 3.2.2 Plaque Index (PI)

Median PI remained stable (baseline: 0.00 [95% CI: 0.03–0.28]; 24 months: 0.33 [95% CI: 0.10–0.57]).

#### 3.2.3 Modified Gingival Index (mGI)

Mean mGI remained stable throughout the study, showing no deviations from baseline (1.45 ± 0.43).

#### 3.2.4 Bleeding on Probing (BOP)

Mean BOP showed no significant change from baseline (0.47 ± 0.29).

#### 3.2.5 Suppuration

Suppuration was observed in two implants at 3 and 6 months and in one implant at 24 months.

#### 3.2.6 Probing Depth (PD)

PD remained stable, ranging from 2.84 ± 0.67 mm at baseline to 3.63 ± 0.95 mm at 6 months.

#### 3.2.7 Peri‐implant Buccal Mucosal Margin (MM)

MM remained coronal to the implant platform, ranging from a median of −2.25 mm (95% CI: −2.71 to –1.13) at 9 months to −0.5 mm at 24 months. At 24 months, the mucosal margin was positioned 1 mm apical to the implant platform in 20% of cases and 2 mm apical in 10%.

#### 3.2.8 Probing Depth to Implant Platform (PD‐IP)

Median PD‐IP ranged from 0.92 mm at 9 months (95% CI: 0.63–1.92) to 2.08 mm at 12 months (95% CI: 0.94–2.75).

#### 3.2.9 Keratinised Tissue

Median KT width remained stable, ranging from 4.00 to 4.50 mm throughout the study.

#### 3.2.10 Mobility

No implants showed mobility during follow‐up.

#### 3.2.11 Radiographic Outcomes

Median bone levels remained stable between baseline and the 24‐month follow‐up (mesial: 2.49–2.97 mm; distal: 2.81–3.17 mm).

#### 3.2.12 Digital Outcomes

**FIGURE 4:**
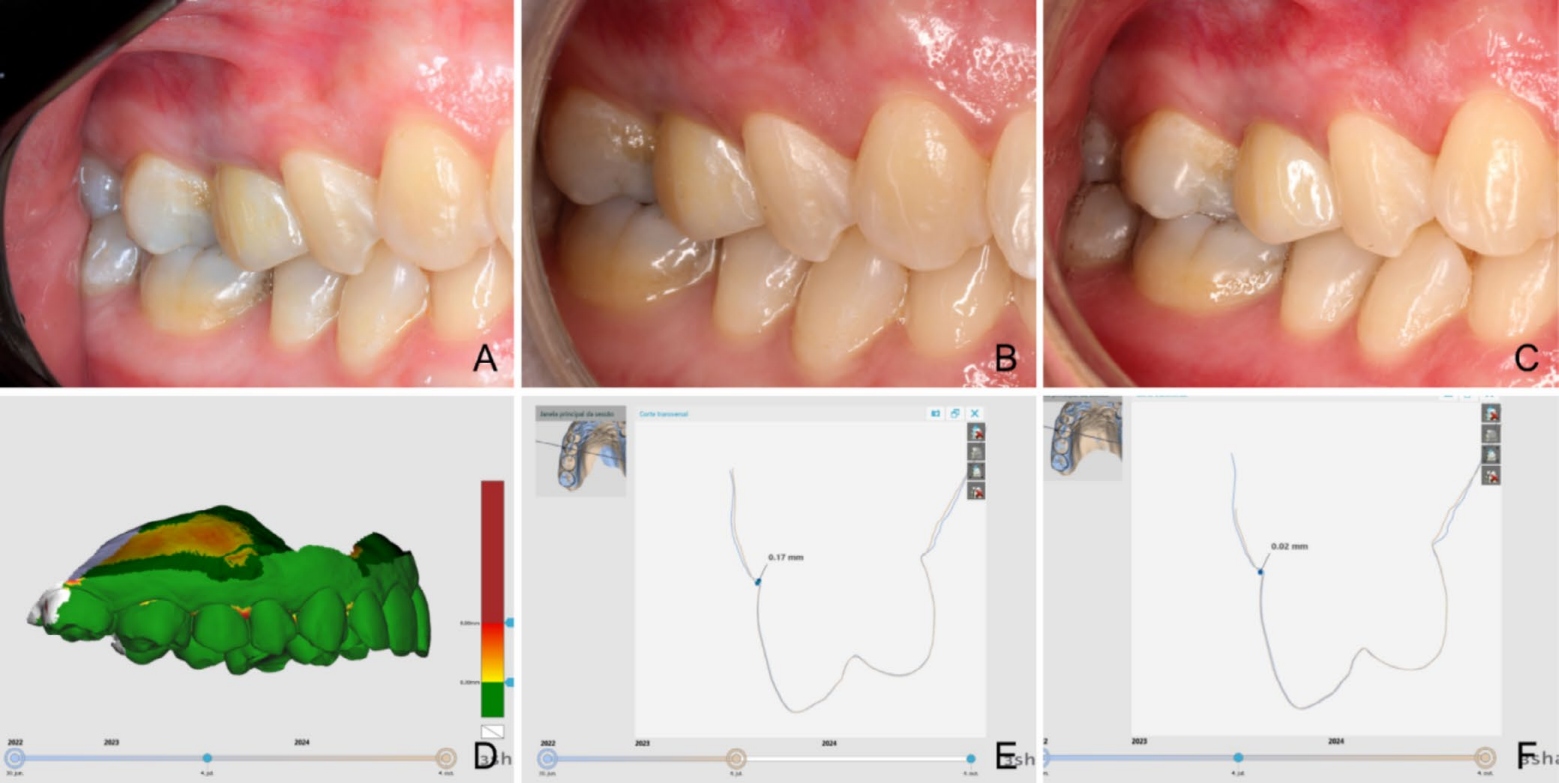
Clinical and digital data of the 1‐ and 2‐year follow‐ups. (A) Representative example of a definitive crown delivery. (B) 1‐year follow‐up. (C) 2‐year follow‐up (D) 3D analysis of intraoral scans, with green colour referring to undetectable changes (E) 1‐year change in mid‐afcial peri‐implant marginal mucosa. (F) 2‐year change in mid‐facial peri‐implant marginal mucosa.

Buccal mucosal change was minimal from baseline (12 months: 0.18 mm [95% CI: 0.11–0.51]; 24 months: 0.20 mm [95% CI: 0.07–0.60]), with no difference between time points (Figure 4).

### 3.3 Patient‐Reported Outcomes

**FIGURE 5:**
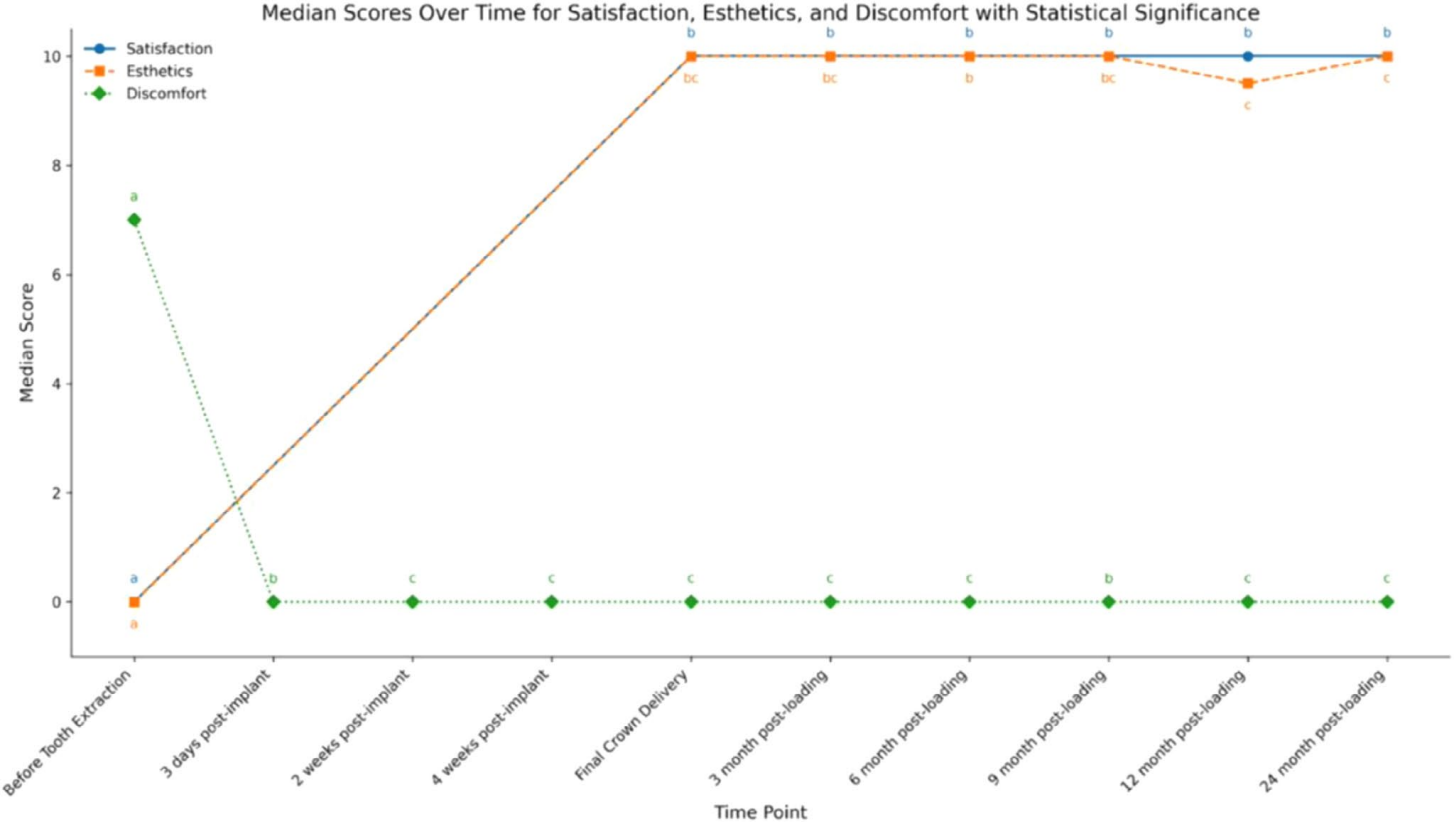
Patient‐reported outcomes. Discomfort, aesthetics and satisfaction VAS scores. Statistical differences are indicated by different letters and were determined using Friedman with a post hoc Durbin–Conover test.

Discomfort significantly decreased from a median of 7.50 (95% CI: 4.86–8.97) prior to extraction to zero (95% CI: 0.11–1.95) at 3 days after implant and remaining at zero thereafter. In parallel, aesthetic satisfaction showed a marked improvement, starting at a median of zero before extraction (95% CI: 00–00), reaching 10 points upon the delivery of the final restoration and sustaining a high median score after that. Additionally, participants reported a high degree of overall satisfaction, with a median score of 10 points from the moment they received the final restoration (95% CI: 10.00–10.00) until the 24‐month follow‐up (95% CI: 9.70–10.10) (Figure 5).

### 3.4 Clinician‐Reported Outcomes

The mean VAS score for the time required for implant placement and provisionalisation was 9.92 ± 0.29. The ease of use received a mean rating of 9.75 ± 0.87 during implant placement and provisionalisation, decreasing to 8.75 ± 1.91 at final restoration delivery. Aesthetic assessment scored 8.83 ± 1.53 at implant placement, 8.50 ± 1.68 at final restoration and 8.10 ± 1.66 at the 24‐month follow‐up.

## 4 Discussion

This study highlights the potential of drill‐free, patient‐specific CAD/CAM‐designed root‐analogue implants for single‐rooted tooth replacement. Over the 24‐month evaluation, these implants exhibited high survival and success rates, along with stable periodontal health, confirming their effectiveness and safety. As a feasibility study of a new technology, it was approved by the Ethics Committee with a sample size limited to 12 participants.

Traditionally, press‐fit implants are placed into pre‐drilled sockets, slightly undersized to ensure a tight fit, often leading to resorption of the bone adjacent to the implant due to excessive compressive forces (Berglundh et al. 2003; Bashutski et al. 2009), failure rates up to 50% (Kohal et al. 2002; Kermalli et al. 2014) and peri‐implant mucosal recession (Monje et al. 2023). This study diverged from conventional methods by employing CBCT, optical impressions, CAD/CAM and additive manufacturing to produce patient‐specific implants. The integration of optical data improved precision, producing implants that fit the alveolar natural socket without additional adjustments, thereby optimising positioning and stability and potentially reducing the bone stress and resorption risks. However, this dual‐data approach required patients to undergo two separate surgical procedures, which could negatively impact their quality of life and extend the overall treatment duration. We recognise that, while effective within the constraints of this study, the current protocol poses limitations to its immediate clinical application. Future developments aimed at streamlining the workflow and integrating planning and placement of patient‐specific implants into a single, efficient procedure are essential to enhance both clinical feasibility and patient experience.

Implants were designed to fit precisely along the mesial‐distal axis and reduce excessive pressure on the buccal bone (Lin et al. 2022). Our clinical, radiographic and digital evaluations indicated stability in the peri‐implant tissues. The stability observed in peri‐implant tissues, particularly on the buccal aspect, evaluated both by clinical exam and using digital tools, mat be attributed to the implant's design and the inclusion criteria, which required an intact buccal bone wall of at least 1 mm thickness (Steigmann et al. 2022). However, even in patients with a thick buccal bone, 20% of implants showed a buccal margin positioned 1 mm apical to the implant platform, and 10% exhibited a mucosal margin 2 mm apically. These findings underscore the importance of continued follow‐up of these cases. Additionally, it is important to recognise that bone healing responses may vary between drilling and non‐drilling implant techniques. Traditional drilling methods trigger a pronounced inflammatory response during the early stages of healing (Salles et al. 2015; Araújo et al. 2015; de Sousa Gomes et al. 2019). While interactions between inflammatory and bone‐forming cells are essential for bone repair and remodelling (Mountziaris et al. 2011), there is evidence that higher levels of acute inflammation are inversely related to the extent of new bone formation at osteotomy sites (Gehrke et al. 2020). Root‐analogue non‐drilling implants are thought to inflict less thermal and mechanical damage, potentially leading to reduced inflammation and more favourable healing outcomes, although further research is needed to confirm this. The success of this drill‐free approach can also be attributed to the meticulous preparation of the alveolar socket. Before implant placement, the socket was thoroughly debrided to remove granulation tissue and immature connective tissue that could hinder osseointegration, while simultaneously promoting bleeding from the bony walls (Araújo et al. 2015; de Sousa Gomes et al. 2019).

The 24‐month results demonstrated 100% survival and 90% success rates, aligning with previous studies reporting survival rates between 80% and 100% for root‐analogue implants manufactured using CBCT data only (Mangano et al. 2014; Moin et al. 2018; Liu et al. 2022). Additionally, the performance of these implants closely mirrored that of immediately placed and loaded single implants (Wittneben et al. 2023). Further supporting these findings, clinical and radiographic evaluations revealed healthy peri‐implant tissues, mirroring results observed from other studies (Mangano et al. 2014; Moin et al. 2018; Liu et al. 2022). Notably, the low incidence of peri‐implant mucositis observed in our study aligns with the diagnostic thresholds proposed by Renvert et al. (2018) and does not conflict with the observed mGI values. An mGI score of 1, reflecting isolated bleeding spots, may not indicate mucositis, as the peri‐implant mucosa exhibits lower resistance to probing than natural gingiva, and, as such, slight bleeding may occur as a response to probing in otherwise clinically healthy sites.

Patients reported significant improvements in comfort and aesthetic satisfaction. Aesthetic scores rose from 0 to 10 upon final restoration, likely due to the stable positioning of the peri‐implant mucosal margin and the minimally invasive nature of the procedure. Clinicians also found the implants to be user‐friendly, time‐efficient and capable of delivering aesthetically pleasing results.

Drill‐free, patient‐specific root‐analogue implants present several advantages, including minimal invasiveness, enhanced patient comfort and a low incidence of postoperative complications. However, their application is most suitable for cases involving atraumatic extractions with intact buccal bony walls, as the feasibility of simultaneous bone grafting with these implants remains uncertain. Additionally, this technique is not suitable for teeth with pronounced root curvatures, which further limits its clinical applicability. These challenges, coupled with the stringent eligibility criteria and a broad, heterogeneous recruitment strategy, contributed to a low inclusion rate. Three intraoperative exclusions resulted from challenges during tooth extraction, which led to bone removal or root fractures. The stringent inclusion and exclusion criteria prioritised patient safety in the first‐in‐human study using this implant but limited the generalisability of findings. Ongoing multi‐centre studies with broader inclusion criteria are under way to assess the clinical applicability and generalisability of this approach.

The limited sample size of this study also restricts the generalisability of our findings. As a feasibility study, our primary objective was to assess the safety and short‐term clinical performance of this novel implant system, rather than to establish its widespread applicability. Larger, adequately powered, randomised controlled trials with broader eligibility criteria are needed to validate these outcomes. Furthermore, the long‐term stability and durability of root‐analogue implants remain unknown, underscoring the need for extended follow‐up to evaluate their long‐term performance. Another limitation pertains to the use of cemented restorations in this study; although effective in demonstrating implant functionality, they are not ideal for routine clinical practice due to the increased risk of complications, such as residual cement‐associated peri‐implantitis. Future studies should explore alternative cement‐free restorative approaches to mitigate these risks.

In conclusion, this study suggests that drill‐free, patient‐specific, one‐piece root‐analogue implants are effective and safe for use in patients, as well as a reliable option for replacing single‐rooted teeth.

## Author Contributions

All authors have made substantial contributions to the conception and design of the study. G.A.R. conceived the project. G.A.R., C.C.V., M.A.H., V.M.S., M.N.C.G.C. and J.D.A. collected the data. R.N. was responsible for the prosthetic phases. C.C.V., M.N.C.G.C. and J.D.A. performed the statistical analysis. G.A.R., C.C.V., V.M.S. and R.N. interpreted the data and wrote the manuscript. M.N.C.G.C. and J.D.A. reviewed the manuscript. All authors approved the version to be published.

## Conflicts of Interest

Giuseppe A. Romito and Cristina C. Villar have received research support from iDENTICAL Inc., Mountain View, California, USA. The other authors declare no conflicts of interest.

## Supporting information


**Figure S1.** Study flow chart. BL = baseline; D = day; M = month; W = week; Y = Year; V = visit.
**Figure S2.** Radiographic assessment. (A) Horizontal cervical line drawn at the level of the implant crown seat. (B) Vertical line measuring implant length (from the crown seat to the implant apex). (C) Vertical line measuring mesial bone level (from the crown seat to the crestal bone on the mesial surface). (D) Vertical line measuring distal bone level (from the crown seat to the crestal bone on the distal surface).

## Data Availability

The data that support the findings of this study are available from the corresponding author upon reasonable request.
