# Supplementary material for: Early Placement of Patient‐Specific One‐Piece Drill‐Free Fully Digital Designed Dental Implants: A Feasibility Study
Source: J Clin Periodontol. 2025 Jul 20;52(10):1374–85. doi: 10.1111/jcpe.14204 (PMC12420077; doi:10.1111/jcpe.14204)
Supplement: Supplementary file 1 — Figure S1. Study flow chart. BL = baseline; D = day; M = month; W = week; Y = Year; V = visit. Figure S2. Radiographic assessment. (A) Horizontal cervical line drawn at the level of the implant crown seat. (B) Vertical line measuring implant length (from the crown seat to the implant apex). (C) Vertical line measuring mesial bone level (from the crown seat to the crestal bone on the mesial surface). (D) Vertical line measuring distal bone level (from the crown seat to the crestal bone on the distal surface). [file JCPE-52-1374-s001.docx]

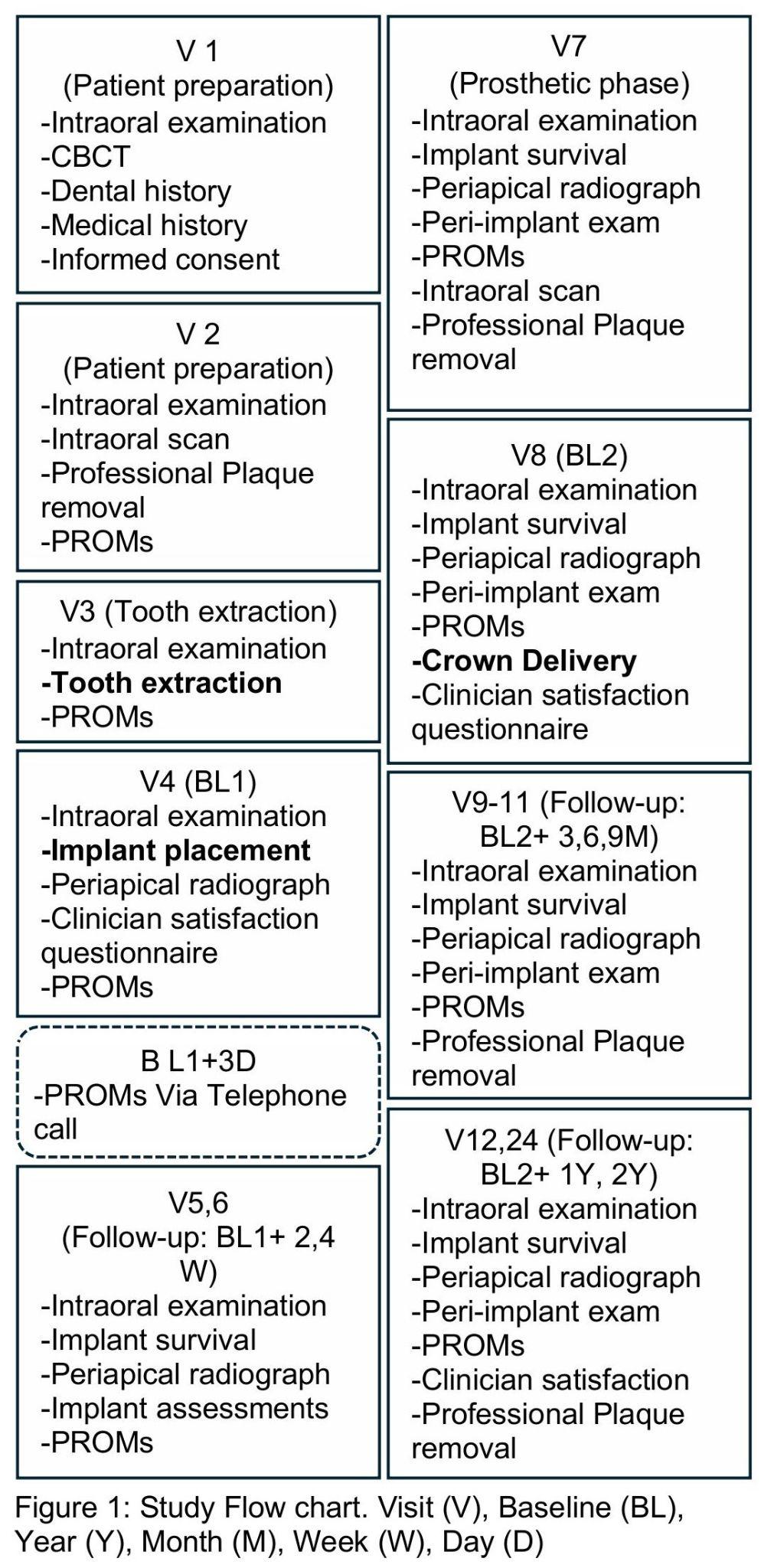


V10 (BL2+

**Supplemental** **Figure 1.** Study flow chart.

BL = baseline; D = day; M = month; W = week; Y= Year; V = visit


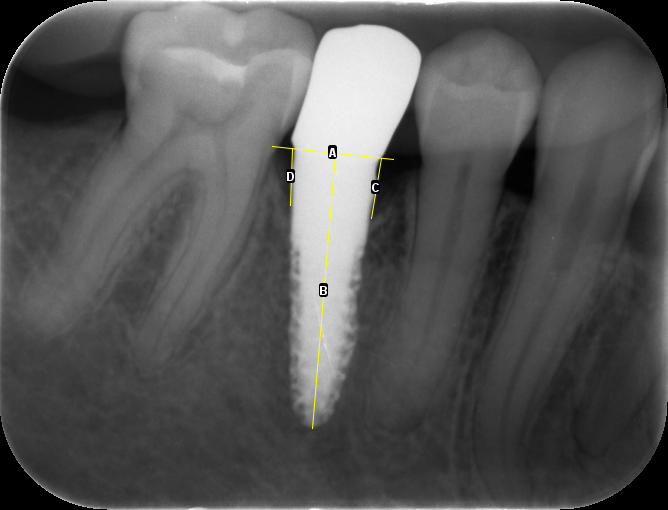


**Supplemental Figure 2.** Radiographic assessment. A: Horizontal cervical line drawn at the level of the implant crown seat. B: Vertical line measuring implant length (from the crown seat to the implant apex). C: Vertical line measuring mesial bone level (from the crown seat to the crestal bone on the mesial surface). D: Vertical line measuring distal bone level (from the crown seat to the crestal bone on the distal surface).
